# cAMP Response Element Binding Protein Is Required for Differentiation of Respiratory Epithelium during Murine Development

**DOI:** 10.1371/journal.pone.0017843

**Published:** 2011-03-08

**Authors:** A. Daniel Bird, Sharon J. Flecknoe, Kheng H. Tan, P. Fredrik Olsson, Nisha Antony, Theo Mantamadiotis, Stuart B. Hooper, Timothy J. Cole

**Affiliations:** 1 Department of Biochemistry & Molecular Biology, Monash University, Clayton, Victoria, Australia; 2 The Ritchie Centre, Monash Institute of Medical Research, Clayton, Victoria, Australia; 3 Department of Medicine, University of Patras, Patras, Greece; Children's Hospital Los Angeles, United States of America

## Abstract

The cAMP response element binding protein 1 (Creb1) transcription factor regulates cellular gene expression in response to elevated levels of intracellular cAMP. *Creb1* ^−/−^ fetal mice are phenotypically smaller than wildtype littermates, predominantly die *in utero* and do not survive after birth due to respiratory failure. We have further investigated the respiratory defect of *Creb1^−/−^* fetal mice during development. Lungs of *Creb1^−/−^* fetal mice were pale in colour and smaller than wildtype controls in proportion to their reduced body size. *Creb1^−/−^* lungs also did not mature morphologically beyond E16.5 with little or no expansion of airway luminal spaces, a phenotype also observed with the *Creb1^−/−^* lung on a *Crem* ^−/−^ genetic background. Creb1 was highly expressed throughout the lung at all stages examined, however activation of Creb1 was detected primarily in distal lung epithelium. Cell differentiation of E17.5 *Creb1* ^−/−^ lung distal epithelium was analysed by electron microscopy and showed markedly reduced numbers of type-I and type-II alveolar epithelial cells. Furthermore, immunomarkers for specific lineages of proximal epithelium including ciliated, non-ciliated (Clara), and neuroendocrine cells showed delayed onset of expression in the *Creb1^−/−^* lung. Finally, gene expression analyses of the E17.5 *Creb1* ^−/−^ lung using whole genome microarray and qPCR collectively identified respiratory marker gene profiles and provide potential novel *Creb1*-regulated genes. Together, these results demonstrate a crucial role for Creb1 activity for the development and differentiation of the conducting and distal lung epithelium.

## Introduction

Survival at birth is critically dependent upon the ability of the lung to immediately take over the role of gas exchange, which in turn, is dependent upon the development of a mature respiratory system during the fetal period. The continuously branching terminal buds of the developing airways contain a population of multipotent epithelial stem or progenitor cells that give rise to the major cell types of the mature airway epithelium [1], [2]. Neuroepithelial, ciliated, and finally non-ciliated secretory Clara cells differentiate within the airway buds that undergo branching morphogenesis during the pseudoglandular stage (∼E10.5–E16.5), while more-distally located alveolar epithelial cells (AECs) begin to differentiate later during the canalicular stage (E16.5–E17.5). Correct differentiation into these cell types, particularly those populating the distal epithelium, is essential to ensure sufficient levels of pulmonary surfactant and surface area for gas exchange, which together are required for survival at birth. The molecular mechanisms regulating differentiation of the lung epithelium into its specialised cell types during development are only partially understood, however it is clear that specific nuclear transcription factors, often acting in complex regulatory networks, play a critical role in mediating this process [3]. In this report we have investigated the role of Creb1 (cyclic adenosine 3′,5′-monophosphate (cAMP) response element binding protein), a downstream transcriptional mediator for a range of systemic signalling factors, in respiratory epithelial differentiation.

Creb1 is a member of the Creb/Atf subfamily of cAMP-responsive basic region-leucine zipper (bZIP) transcription factors which include activating transcription factor 1 (Atf1) and the cAMP response element modulatory protein (Crem) [4]. A large range of signalling factors including steroid hormones, peptide hormones, growth factors and other cytokines can induce activation of Creb1, usually via stimulation of pathways which increase intracellular levels of cAMP. Elevated levels of cAMP activate and release catalytic subunits of protein kinase A (PKA) which phosphorylate Creb1 primarily at Ser133 [5], [6], [7]. Once phosphorylated, Creb1 can bind as homo- or heterodimers with other Creb/Atf subfamily members to cAMP response elements or CREs (‘TGACGTCA’) in promoter regions and transactivate specific target genes [4].

Gene targeted mutations of the Creb/Atf1 subfamily in mice have provided important insight into the biological function of these factors during development. Mice lacking *Atf1* and *Crem* develop normally, although *Crem* ^−/−^ males are sterile due to defective spermatogenesis [8], [9]. Inactivation of *Creb1* isoforms α and Δ (*Creb1* ^αΔ^) produce normal, viable mutant offspring, however these mice are found at a reduced mendelian frequency at birth suggesting a developmental disadvantage in utero [10]. On the other hand, combined deletion of *Creb1* isoforms α, β and Δ (*Creb1* ^−/−^) causes a complete loss of *Creb1* function, leading to perinatal death [11]. Investigation of Creb/Atf1 subfamily mouse mutants has also revealed that Creb/Atf1 subfamily members may compensate for one another. Upregulation of Crem expression has been noted in many organs of *Creb1* ^αΔ^ and *Creb1* ^−/−^ mice, particularly in the brain [10], [11], [12]. Furthermore, *Atf1* ^−/−^ *Creb1* ^−/−^ and *Atf1* ^+/−^ *Creb1* ^−/−^ double transgenic mice die early in development indicating that Creb1 and Atf1 may also compensate for each other [9].

The role of the Creb/Atf1 subfamily in lung development however has not been well described. To further investigate the role of Creb1 in the developing lung, we analysed the lung phenotype of *Creb1^−/−^* fetal mice in detail from E15.5 till just before birth at E18.5. We firstly show that Creb is required for morphological development of the lung beyond E16.5. Importantly, we also demonstrate that any possible compensatory effect caused by up-regulation of *Crem* in the absence of Creb1 does not alter the *Creb1* ^−/−^ lung phenotype. We found that although Creb is widely expressed in the lung it is activated primarily in the distal epithelium. Given this, we investigated whether differentiation of cells within the distal epithelium was adversely affected. Differentiation of cell types from more proximal conducting airway epithelium such as non-ciliated (Clara), ciliated and neuroendocrine lineages was also examined in the *Creb1^−/−^* lung. Finally, gene expression analyses of E17.5 *Creb1* ^−/−^ lungs using whole genome microarray and quantitative PCR (qPCR) was used to identify respiratory marker gene profiles in order to provide potential novel *Creb1*-regulated genes. Together, our results point toward a crucial role for Creb1-mediated signalling in the differentiation of both the developing distal and proximal lung epithelium.

## Materials and Methods

### Mice

All animal experimentation was approved by the School of Biomedical Sciences Animal Ethics Committee, Monash University (Ethics no. 2009/63), and was carried out according to the National Health and Medical Research Council of Australia guidelines for the breeding, care and use of genetically modified and cloned animals for scientific purposes 2007. *Creb1^lox^* ^P/*lox*P^ mice on a C57Bl/6 genetic background were generated by gene-targeting as described previously [12]. These mice were bred with mice harbouring a neural progenitor specific- (nestin) driven Cre recombinase to produce *Creb1^Nescre^* mice. A small proportion of the *Creb1^Nescre^* mice showed unexpected Cre activity in germline cells, which led to the production of fully heterozygous *Creb1^+^* ^/−^ mice where the mutated allele contained a Cre-mediated deletion of exon 10 of the mouse *Creb1* gene [12]. Timed-mated *Creb1^+^* ^/−^ mice were sacrificed at E15.5 to E18.5 p.c. (post coitum) by cervical dislocation for subsequent collection of fetal lungs. Timed matings of *Creb1^+^* ^/−^ mice on a *Crem* ^−/−^ genetic background were also peformed to obtain *Creb1^−^* ^/−^, *Crem* ^−/−^ double transgenic fetal mice. Tail snips were genotyped at the *Creb1* and/or *Crem* gene locus by PCR.

### Histology and immunohistochemistry

Lung tissue was fixed in 4% paraformaldehyde overnight, processed for paraffin embedding, and 5 µm paraffin sections prepared for analysis. Sections were incubated at 60°C for 2 hours and then deparaffinised using three washes of xylene, hydrated with 3 washes of 100% ethanol, then tap water and stained with haematoxylin/eosin for histological analyses. For immunohistochemical analyses, sections were hydrated using several washes of 100%, 95% and 70% ethanol and finally dH_2_O. For immunostaining procedures, antigen retrieval was performed by microwaving slides in 10 mM sodium citrate for 20 minutes. Endogenous peroxidases were quenched with 3% H_2_O_2_ in methanol. Sections were treated with either specific animal serum or 2% Bovine Serum Albumin (BSA) to block non-specific binding and incubated with the following primary antibodies: Ki67 (Labvision, Fremont, CA), ProSPC (Chemicon, Temecula, CA), Sox9 (kindly provided by Ass. Prof Vince Harley, Prince Henry's Institute, Melbourne), SPD (Abcam, Cambridge, UK), Scgb1a1 (Santa Cruz, CA), Creb1 (Cell Signaling, Danvers, MA) pCreb1 (Cell Signaling), CGRP (Calibiochem, La Jolla, CA), FoxJ1 (ABR, Golden, CO), CD31 (Abcam), αSMA (Abcam), washed 3 times in PBST (PBS with 0.1% Tween-20) then incubated with biotinylated secondary antibodies, (either rabbit anti-goat, Zymed, San Francisco, CA, goat anti-rabbit, Vector laboratories, Burlingame, CA or rat anti-mouse IgG1, Invitrogen, Carlsbad, CA). In the case of SPD, tryamide signal amplification (Perkin Elmer, Waltham, MA) was used to enhance signal strength. Sections were again washed in PBST and the biotinylated secondary antibody detected using LSAB®2 Streptavidin-HRP (Dako, Glostrup, Denmark) and diaminobenzidine (DAB) (Dako) systems as per manufacturer's instructions and finally counterstained with haematoxylin. Sections were also treated with antibody diluent (no primary antibody) and secondary antibody to serve as a negative control.

### Cell proliferation index and TUNEL analysis

Sections immunostained with Ki67/haematoxylin (as described above) were viewed using a light microscope and images from proximal and distal regions of the fetal lung from three E18.5 *Creb* ^−/−^ and three E18.5 wildtype mice were collected. For cell counting analyses, at least 1000 cells were counted per animal using multiple sections and fields of view per section. Fields of view were carefully chosen to avoid counting the same cell twice or more. Numbers of immuno-positive cells (brown from HRP-DAB reaction) were compared to the number of immuno-negative cells (purple from haematoxylin histological stain) to determine a labeling index. Terminal deoxynucleotidyl transferase-mediated dUTP-biotin nick end-labelling (TUNEL) was performed using an ApopTag Plus Peroxidase In Situ Detection Kit (Chemicon International, Temecula, CA) as per manufacturer's instructions. Briefly, hydrated fetal lung sections (see above) were treated with Proteinase K (20 g/mL), washed in distilled water, and then treated with 3% hydrogen peroxide to quench endogenous peroxidases. The sections were then washed again in distilled water, treated with terminal deoxynucleotidyl transferase, before the reaction was stopped by incubation in stop/wash buffer. Sections were washed in PBS and then treated with anti-digoxignenin conjugate at room temperature. After washing again in PBS, sections were treated with peroxidase substrate to visualize staining, washed in distilled water and counterstained with Nuclear Fast Red. Slides were dehydrated and mounted (as above).

### Isolation of total fetal lung RNA

Total RNA was prepared from fetal lung by homogenization in TRIzol reagent (Gibco/BRL, Rockville, MD), a guanidinium isothiocyanate/phenol-based solution. Following chloroform extraction, RNA was precipitated with isopropanol, washed in 70% ethanol and redissolved in water. For microarray analysis, total RNA was further purified using the Purelink™ Micro-to-Midi™ Total RNA Purification System (Invitrogen).

### Synthesis of cDNA and Quantitative Real-Time PCR

qPCR was performed using cDNA prepared from total lung RNA as described above and reverse transcribed into cDNA using random hexamers and M-MLV Reverse Transcriptase, RNase H Minus, Point Mutant (Promega, Madison WI). Oligonucleotide primer pairs for qPCR analyses were designed using the web-based Primer3 software. cDNAs were assayed in triplicate and levels of 18S rRNA was used as a normalising control. Primer sequences were (5′ to 3′): *Atf1*, forward, AGACCTACCAGATCCGTACCA, reverse, GTCATCACCACGGTCTGC; *Crem*, forward, CGAGGTCCGCTACGTAAAC, reverse, CTTCGATCCTGCTGTGATTC; *Sftpa*, forward, AATGGGAGTCCTCAGCTTG, reverse, ACTGACTGCCCATTGGTG; *Sftpa*, forward, CAGGTGCAGCTATCACGTC, reverse, GCTTTGGCACCAGAATTG; *Sftpc*, forward, GAGTCCACCGGATTACTCG, reverse, GATGAGAAGGCGTTTGAGG; *Sftpd*, forward, TCATGTGTAGCCCAACAGAG, reverse, AAACCTGGATCACCCTTCTC; *Abca3*, forward, ACTTCTATGCACAGCTGAAAGG, reverse, TTCCTCAGGACACTTCTGGA; *Aqp5*, forward, CTGCGGTGGTCATGAATC, reverse, CTACCCAGAAGACCCAGTGA; *Foxj1*, forward, AAGGCCACCAAGATCACTC, reverse, ATGGAATTCTGCCAGGTG; *Scgb1a1*, forward, GCTCAGCTTCTTCGGACA, reverse, CAGACTCTGATTCCATGAGGA; *Calca*, forward, GAGGGCTCTAGTGTCACTGC, reverse, GTTGTCCTTCACCACACCTC; *Chi3l1*, forward, GACCAGAAACACCAACCTGA, reverse, CCATCAAAGCCATAAGAACG; *Lyz1/2*, forward, GCTGACTGGGTGTGTTTAGC, reverse, TCCACGGTTGTAGTTTGTAGC; *Lcn2*, forward, GGAAATATGCACAGGTATCCTC, reverse, AAATACCATGGCGAACTGG; *Hist2h3c1*, forward, CTTGCCTTAGTTAACACCCTCA, reverse, TTCAAGATTCTCCTTCTAGATTGTTAC; *Hist1h3g*, forward, CTACCTCGTGGGTCTGTTTG, reverse, CAGAAACCCTTAAGCCCTCT; *D6Mm5e*, forward, TGTGCAAGTGAGAGGGAACT, reverse, GCTGGAGCCCATCTTCTATT. Cycling was performed using Platinum® SYBR® Green qPCR SuperMix (Invitrogen) on a Rotor-Gene™3000 (Corbett Research, Sydney, NSW). qPCR data was analysed using RotorGene 6.0 software (Corbett Research, Sydney, Australia) and differential expression determined using the comparative delta-delta CT method [13].

### Analysis of AEC phenotypes

For the purposes of transmission electron microscopy, the entire right lung of E17.5 *Creb1* ^−/−^ (*n* = 5) and wildtype (*n* = 4) fetal mice was post-fixed in 2% glutaraldehyde [14]. The lung was then cut into either two or more parts (depending on overall lung size), avoiding major airways and blood vessels. Tissue was washed in 0.1 M cacodylate buffer, incubated in 2% OsO_4_ (in 0.1 M cacodylate buffer), dehydrated in a series of ethanol and propylene oxide washes and embedded in epoxy resin. Between two and three epoxy resin/tissue blocks were randomly chosen from each animal, ensuring that samples were taken from both the upper and lower regions of each lung. Ultra thin sections (70–90 nm) were cut using a diamond knife, stained with aqueous uranyl acetate and Reynolds lead citrate and coded for blinded analysis. Alveolar epithelial cells were identified using a transmission electron microscope (Joel 100 s). For each animal, a minimum of 100 AECs were classified and the proportions of each phenotype determined by counting the number of nuclear profiles of each type [14], [15]. Identification of AECs depended on clear visualization of the basement membrane with all AECs localised on the luminal surface of this membrane. AECs were categorized as one of four phenotypes; undifferentiated AECs, type-I AECs, type-II AECs and intermediate AECs according to strict morphological criteria. Undifferentiated AECs were identified by their abundant cytoplasmic glycogen. Type-I AECs had flattened cytoplasmic extensions, flattened nuclei, little perinuclear cytoplasm and few cytoplasmic organelles. Type-II AECs were rounded in shape with a rounded nucleus, had microvilli on their apical surface and abundant cytoplasmic organelles, including lamellar bodies. Numbers of lamellar bodies per cross-sectional area of each type-II AEC were also quantified. The intermediate cells were a heterogenous group that displayed characteristics of both type-I and type-II AECs; these cells have cytoplasmic extensions usually associated with type-I AECs but also exhibit type-II AEC characteristics such as lamellar bodies and apical surface microvilli [14], [15], [16], [17], [18].

### Microarray gene expression analysis

Whole genome expression microarray analysis was performed by the Australian Genome Research Facility with total RNA from E17.5 *Creb1* ^−/−^ and wildtype fetal lung (*n* = 4). 3 ug of RNA was subjected to rRNA removal procedure using RiboMinus Human/Mouse Transcriptome Isolation Kit (Invitrogen) and subsequently labelled using the Affymetrix WT cDNA Amplification kit (Millenium Sciences). Single stranded cDNA was end-labelled using terminal deoxynucleotidyl transferase enzyme from the WT Terminal Labelling kit (Millenium Sciences). Labelled cDNA was then hybridised to the Affymetrix *MouseExon 1.0 ST Array* GeneChip (Millenium Sciences cat no. 900817). Chips were hybridized at 45°C for 16 hours, washed using the FS450_0001 (49 array format) fluidics script in the Fluidics Station 450 (Affymetrix), and scanned using the GeneChip Scanner 3000450 (Affymetrix). The scanner operating software, GCOS, converted the signal on the chip into a DAT file, which was used for generating CEL files for analysis. The affymetrix CEL files were imported into Partek® Genomics Suite software package, version 6.3, build 6.08.0205 Copyright © 2007 (Partek Inc., St. Louis, MO, USA.) and the analysis was performed using the predefined workflow for gene expression analysis. Expression values were background corrected using the GCRMA algorithm (Robust Multi-array analysis with correction for GC content) [19], [20]. Data were normalized [21] and summarized using Median Polish algorithm. Principal Component Analysis was used to transform gene expression information into variance-based information. The normalized data were then subjected to ANOVA model using the Bonferroni method [22]. The False discovery rate (FDR) [23] was calculated based on the *p*-value (≤0.01) from ANOVA. Genes that experienced a change of 1.5 fold or more in signal intensity and FDR of less than 0.05 were considered to be differentially expressed. The raw intensity data of each gene was used for the calculation of a “z-score”. Z-scores for each gene were normalized and expressed as a unit of a standard deviation from the normalized mean of zero. All Affymetrix-microarray data presented in this manuscript is MIAME compliant and has been submitted to the European Bioinformatics Institute ArrayExpress repository, Cambridge, UK. The data can be viewed by accessing the following web site: http://www.ebi.ac.uk/microarray-as/aer/login and using the following information: Login: Reviewer_E-MEXP-1295, Password: 9achuSeC, Accession number E-MEXP-1295.

### Statistical Analysis

The statistical significance of the cell proliferation index, qPCR expression profiles, AEC and lamellar body indexes were all determined by a non-paired Student's *t*-Test with statistical significance set at *p*<0.05 and error bars depicting standard error of the mean (SEM).

## Results

### Morphological defects in lungs of *Creb1* ^−/−^ mice are first detected after E16.5

**Figure 1:**
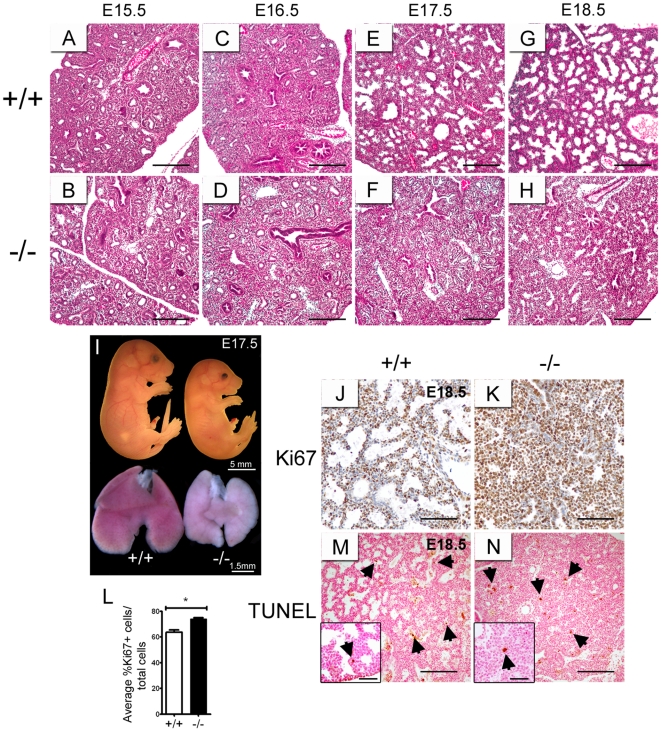
Morphological analysis of lung development in lungs of *Creb1* ^−/−^ mice. Haematoxylin and eosin-stained tissue sections from E15.5–E18.5 *Creb1* ^−/−^ and wildtype lungs (A–H). Lung morphology of *Creb1* ^−/−^ and wildtype fetal mice was similar at E15.5 (A, B) and E16.5 (C, D). At E17.5 proximal and distal airways of *Creb1* ^−/−^ mice failed to expand and showed compacted tissue morphology (F). Lungs of E18.5 *Creb1* ^−/−^ mice showed a comparable morphology to E17.5 *Creb1* ^−/−^ lungs (H). In comparison littermate controls at E17.5–E18.5 showed normal airway expansion (E, G). Lungs of E17.5 *Creb1* ^−/−^ mice were smaller than wildtype, though in approximate proportion to a reduced body size of *Creb1* ^−/−^ mice (I). Immunohistochemical analysis for the cell division marker Ki67 in lungs of E18.5 wildtype (J) and *Creb1* ^−/−^ (K) mice. Quantification of Ki67-positive and -negative cells (*n* = 3) in total lung showed a small increase in cell proliferation in lungs of *Creb1* ^−/−^ mice at E18.5 (L). TUNEL analysis for apoptotic nuclei showed very rare, but comparable numbers of apoptotic cells in E18.5 wildtype (M) and *Creb1* ^−/−^ (N) lungs (arrows indicate apoptotic nuclei). Error bars represent SEM. Asterisk (*) indicates *p*<0.05. Scale bars: *A–H*, 200 µm; *I*, 5 mm for whole pups and 1.5 mm for fetal lungs as shown; *J,K*, 100 µm; *M*,*N,* 200 µm; insets for *M* and *N*, 20 µm.

Haematoxylin and eosin-stained fetal lung tissue sections of E15.5 to E18.5 *Creb1^−/−^* and wildtype mice were analysed for gross morphology using light microscopy. Lungs from E15.5 and E16.5 *Creb1^−/−^* mice showed similar tissue morphology to wildtype with respect to proximal and distal airway structure (Fig. 1A–D). At E17.5 and E18.5, the lungs of wild type mice showed the expected progressive expansion of the terminal airsacs indicative of the normal transition from the pseudoglandular stage to the canalicular-saccular stages (Fig. 1E, G). In comparison, the lungs of E17.5 and E18.5 *Creb1* ^−/−^ mice displayed little expansion of both the proximal and distal airways and showed a gross morphology comparable to E15.5–E16.5 lungs. The lack of round terminal saccular structures and the predominance of elongated tubules indicate an arrest or delay of normal lung development (Fig. 1F, H). The proportion of *Creb1^−/−^* mice which were present in each litter at the stages collected was also determined. *Creb1^−/−^* mice were found at 12.7% (E15.5), 9.0% (E16.5), 6.7% (E17.5) and 8.7% (E18.5) (Table S1). Interestingly, these proportions are lower than those found in a different strain of *Creb1^−/−^* mice, which showed a 15% rate at birth [11].

Intact *Creb1^−/−^* and wildtype fetal mice and their lungs were also visualised at E17.5 when the developmental defect was first observed. Lungs from *Creb1^−/−^* mice were paler in colour and noticeably smaller than wildtype although this was proportional to the smaller size (30%) of the *Creb1^−/−^* pup (Fig. 1I). Creb/Atf1-mediated signalling has been strongly implicated as a regulator of both cell proliferation [24] and apoptosis [9] during development. Therefore we investigated whether a loss of cell proliferation or perhaps increased apoptosis was the cause of the reduction in size of the *Creb1^−/−^* lung. Sections from E18.5 *Creb1^−/−^* and wildtype lungs were immunostained for Ki67 (Fig. 1J,K), a nuclear marker of cell division, and also assessed by a TUNEL analysis (Fig. 1M,N), which detects apoptotic nuclei. The average percentage of Ki67-postive cells however, was only slightly higher (1.2 fold, *p*<0.05, Fig. 1L) in the *Creb1^−/−^* lung relative to wildtype, indicating that lack of cell proliferation is not a contributing factor to the small size of the *Creb1^−/−^* lung. Apoptotic cells were detected at levels too small for a statistical analysis however there seemed to be no obvious differences between wildtype (Fig. 1M) and *Creb1* ^−/−^ (Fig. 1N) lung sections, indicating that increased apoptosis is also not a contributing factor toward the small size of the *Creb1^−/−^* lung.

### Compensatory up-regulation of *Crem* does not alter *Creb1* ^−/−^ morphology in the developing lung

**Figure 2:**
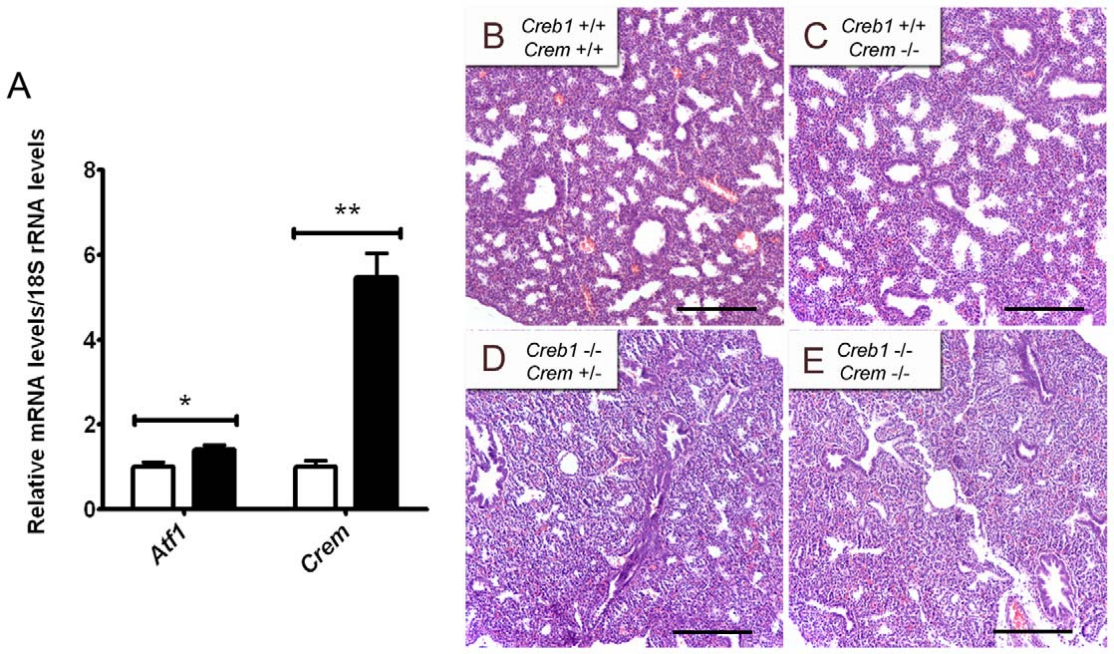
Compensatory up-regulation of *Crem* does not alter *Creb1* ^−/−^ morphology in the developing lung. qPCR analysis of *Crem* and *Atf1* mRNA levels in the E17.5 *Creb1* ^−/−^ and wildtype lung (*n* = 4) (A). Haematoxylin and eosin-stained tissue sections from E17.5 lungs of *Creb1* ^+/+^, *Crem* ^+/+^ (B), *Creb1* ^+/+^, *Crem* ^−/−^ (C), *Creb1* ^−/−^, *Crem* ^+/−^ (D) and *Creb1* ^−/−^, *Crem* ^−/−^ (E) mice. Lungs of E17.5 *Creb1* ^−/−^, *Crem* ^−/−^ mice show no morphological differences as compared to *Creb1* ^−/−^, *Crem* ^+/−^ mice. Error bars represent SEM. Asterisk (*) indicates *p*<0.05 and ** indicates *p*<0.001. *White bars*: Wildtype, *Black bars: Creb1* ^−/−^. Scale bars: 100 µm.

Previous findings have shown that expression of Crem is upregulated in many organs in the absence of Creb1 [10], which may complicate the analysis of the *Creb1* ^−/−^ phenotype. Using qPCR analysis, we found that levels of *Crem* and also *Atf1* mRNA were increased 5.5 fold (*p*<0.001) and 1.4 fold (*p*<0.05) respectively in lungs of E17.5 *Creb1* ^−/−^ mice (*n* = 4) relative to wildtype (Fig. 2A). We therefore examined lungs of E17.5 *Creb1* ^−/−^, *Crem* ^−/−^ double transgenic mice and looked for potential morphological defects in addition to the already-described defects seen in the E17.5 *Creb1* ^−/−^ lung. In comparison to *Atf1* ^−/−^, *Creb1* ^−/−^ and *Atf1* ^+/−^, *Creb1* ^−/−^ double transgenic mice [9], *Creb1* ^−/−^, *Crem* ^+/−^ and *Creb1* ^−/−^, *Crem* ^−/−^ double transgenic mice developed normally suggesting that Creb1 and Crem do not act in concert to fulfil an essential role during embryogenesis (data not shown). Lungs from E17.5 *Creb1* ^−/−^, *Crem* ^+/−^ (Fig. 2D) and *Creb1* ^−/−^, *Crem* ^−/−^ (Fig. 2E) mice also showed no overt morphological differences to the phenotype described for *Creb1* ^−/−^, *Crem* ^+/+^ mice (Fig. 1F). To determine any differences in the lung caused by lack of *Crem* alone, we also analysed lung morphology in E17.5 *Crem* ^−/−^ mice. As expected, lungs from *Crem* ^−/−^ mice showed no obvious defects at E17.5 (Fig. 2C) in accordance with the normal postnatal survival phenotype of these mice [8]. Together, this suggests that the up-regulation of *Crem* in the lung of *Creb1* ^−/−^ mice has no effect on the respiratory phenotype.

### Creb1 is activated primarily in the distal lung epithelium during late gestation

**Figure 3:**
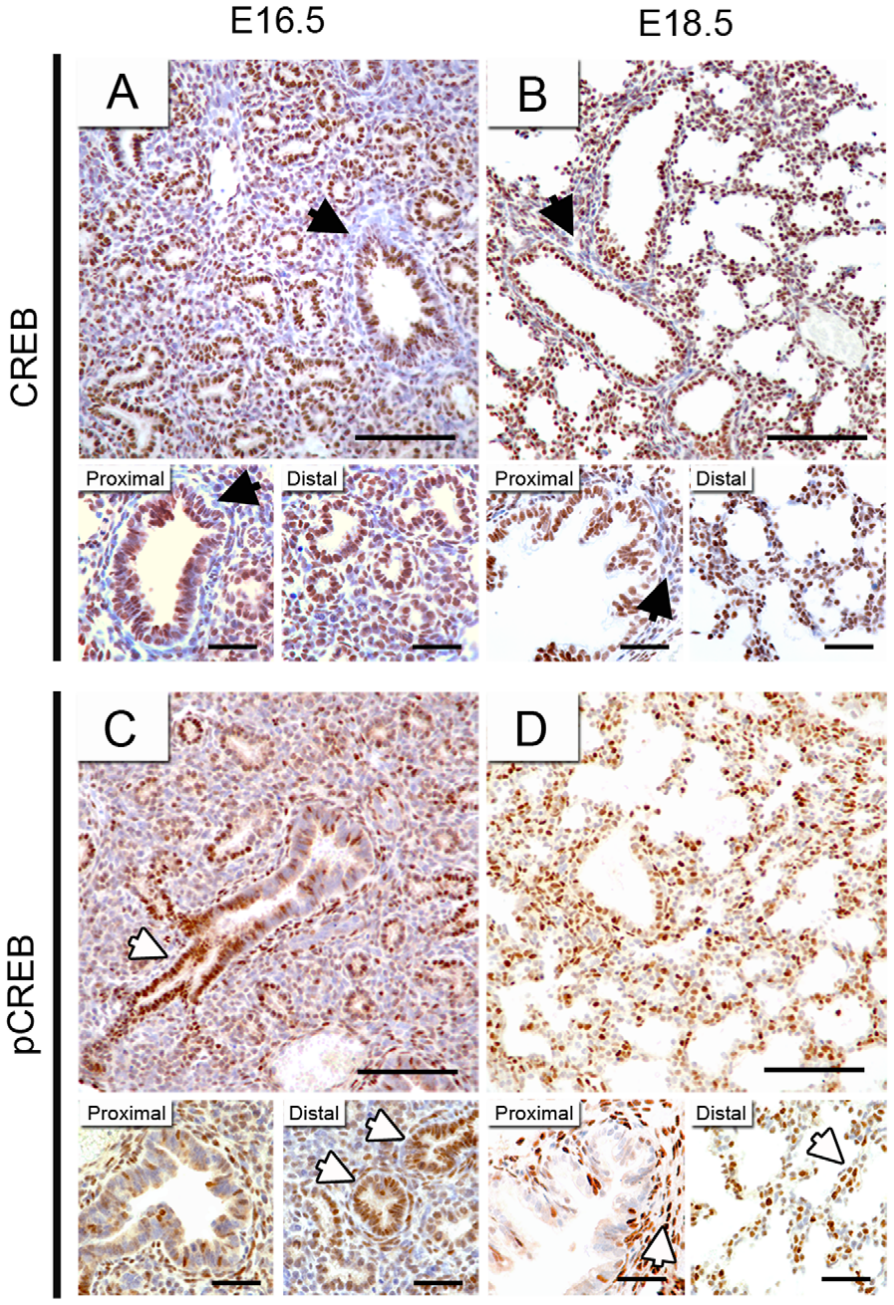
Creb1 is activated primarily in the distal lung epithelium during late gestation. Immunohistochemistry for Creb1 (A,B) and pCreb1 (C,D) on tissue sections of E16.5 and E18.5 wildtype lungs. Creb1 expression was detected in most cells at E16.5 (A) and E18.5 (B), though a proportion of mesenchymal cells surrounding large airways were Creb1-negative (Black arrows). At E16.5, pCreb1 staining was detected sparsely in the mesenchyme and proximal epithelium but in most cells of the distal epithelium (C, white arrows). At E18.5, pCreb1 staining was found primarily in the mesenchyme surrounding large airways and also within saccular walls (D, white arrows). Scale bars: upper panels, 100 µm; magnified lower panels of proximal and distal lung, 25 µm.

Using immunohistochemistry, we determined the localisation of Creb1 protein at E16.5 and E18.5, timepoints which occur before and after the onset of the lung defect at E17.5 observed in *Creb1* ^−/−^ mice. Creb1 protein was detected in the nuclei of most cells in the E16.5 and E18.5 wildtype lung. However, while epithelial cells from both proximal and distal lung almost always showed Creb1 expression a large number of mesenchymal cells showed no Creb1 immunoreactivity, particularly surrounding large airways (Fig. 3A, B). We also performed Creb1 immunohistochemistry on sections of *Creb1* ^−/−^ lungs to validate our mouse knockout model. At E18.5, Creb1 protein was strongly detected in wildtype lung (as previously mentioned), however in lung tissue of E18.5 *Creb1* ^−/−^ mice no Creb1 immunoreactivity could be seen (Fig S1).

As the transcriptional activation of Creb1 via cAMP is dependent upon specific phosphorylation at Ser133 [7], we analysed the presence of activated Creb1 (pCreb1) using a phospho-Ser133 Creb1 antibody. In E16.5 lung, pCreb1 was detected sporadically in almost all cell types of the developing lung, however cells from the distal epithelium almost always showed pCreb1 expression (Fig. 3C). In contrast, at E18.5 pCreb1 expression in distal lung epithelium was much reduced, while more expression was seen in the interstitial mesenchyme (Fig. 3D). Increased levels of mesenchymal staining were also observed surrounding the proximal epithelium (Fig. 3D).

### Vascular development is unaffected in lungs of *Creb1* ^−/−^ mice

**Figure 4:**
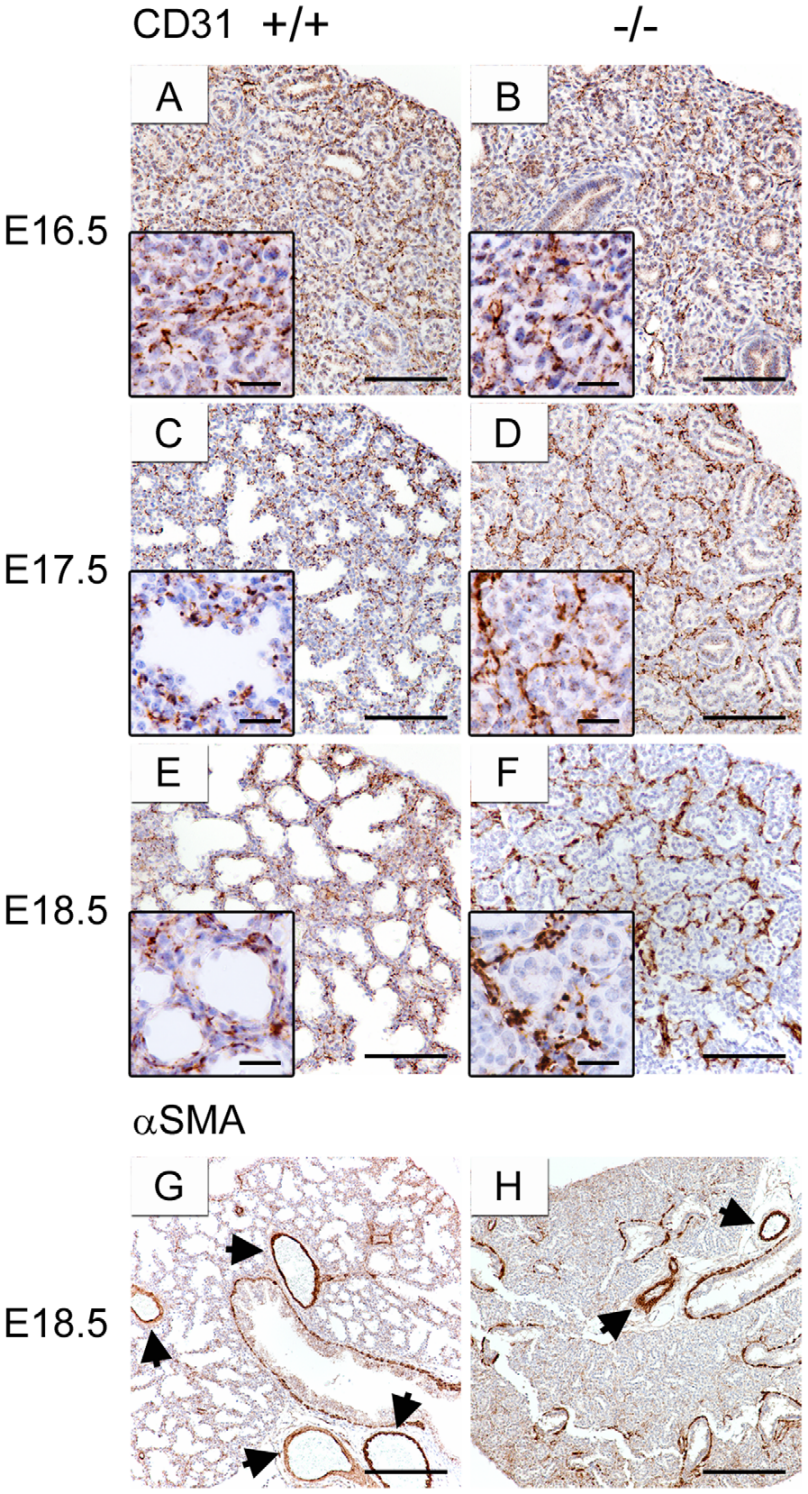
Vascular development is unaffected in lungs of *Creb1* ^−/−^ mice. Immunohistochemistry for CD31 (A–F) and αSMA (G,H) in the lung of E16.5 to E18.5 *Creb1* ^−/−^ and wildtype mice. In wildtype mice, CD31 immunostaining shows a normal vascular structure with large blood vessels and in capillaries in the distal lung (A,C,E). Vascular structure in the lung of E16.5 *Creb1* ^−/−^ mice appears similar to E16.5 wildtype mice (B). An intact, although immature vasculature is seen in the lung of E17.5 (D) and E18.5 (F) *Creb1* ^−/−^ mice. A normal smooth muscle layer surrounding large blood vessels (arrows) was also detected in both E18.5 wildtype (G) and E18.5 *Creb1* ^−/−^ (H) mice. Scale bars: 100 µm; insets, 20 µm.

Since we observed that intact lungs of *Creb1* ^−/−^ fetal mice were paler than wildtype controls (Fig. 1I), and also that Creb1 and pCreb1 expression were often seen in pulmonary endothelial cells (data not shown), we reasoned that loss of Creb1 may impair pulmonary circulation via improper development of the lung vasculature. To investigate this, lung sections of E16.5 to E18.5 wildtype and *Creb1* ^−/−^ mice were stained for the endothelial marker CD31 (also known as Pecam1) using immunohistochemistry. In wildtype mice, CD31 staining showed the expected pattern of pulmonary vasculature development throughout the lung with normal appearance of large blood vessels and an extensive capillary network surrounding the distal epithelium (Fig. 4A,C,E). In lungs of E16.5 *Creb1* ^−/−^ mice, the vasculature structure appeared similar to E16.5 wildtype mice (Fig. 4B). At E17.5 and E18.5 the vasculature structure seemed disorganised in lungs of *Creb1* ^−/−^ mice relative to wildtype controls, and retained an immature morphology similar to E16.5 wildtype lungs (Fig. 4D,F). However it was also evident that the endothelial network remained intact in E17.5 and E18.5 *Creb1* ^−/−^ lungs with evidence of large blood vessels and also capillaries in close proximity to the epithelium. We also stained wildtype and *Creb1* ^−/−^ lung sections at E17.5 for the smooth muscle cell marker, alpha smooth muscle actin (αSMA). In wildtype mice αSMA staining showed a normal layer of smooth muscle surrounding large airways and blood vessels (Fig. 4G). Lungs of *Creb1* ^−/−^ mice also showed a normal layer of smooth muscle cells surrounding large airways and blood vessels (Fig. 4H). Together this suggests that lack of Creb1 does not directly impair gross development of the pulmonary vasculature, although structural organisation of the microvasculature appears altered at E18.5.

### Defective AEC differentiation and lamellar body development in lungs of *Creb1* ^−/−^ mice

**Figure 5:**
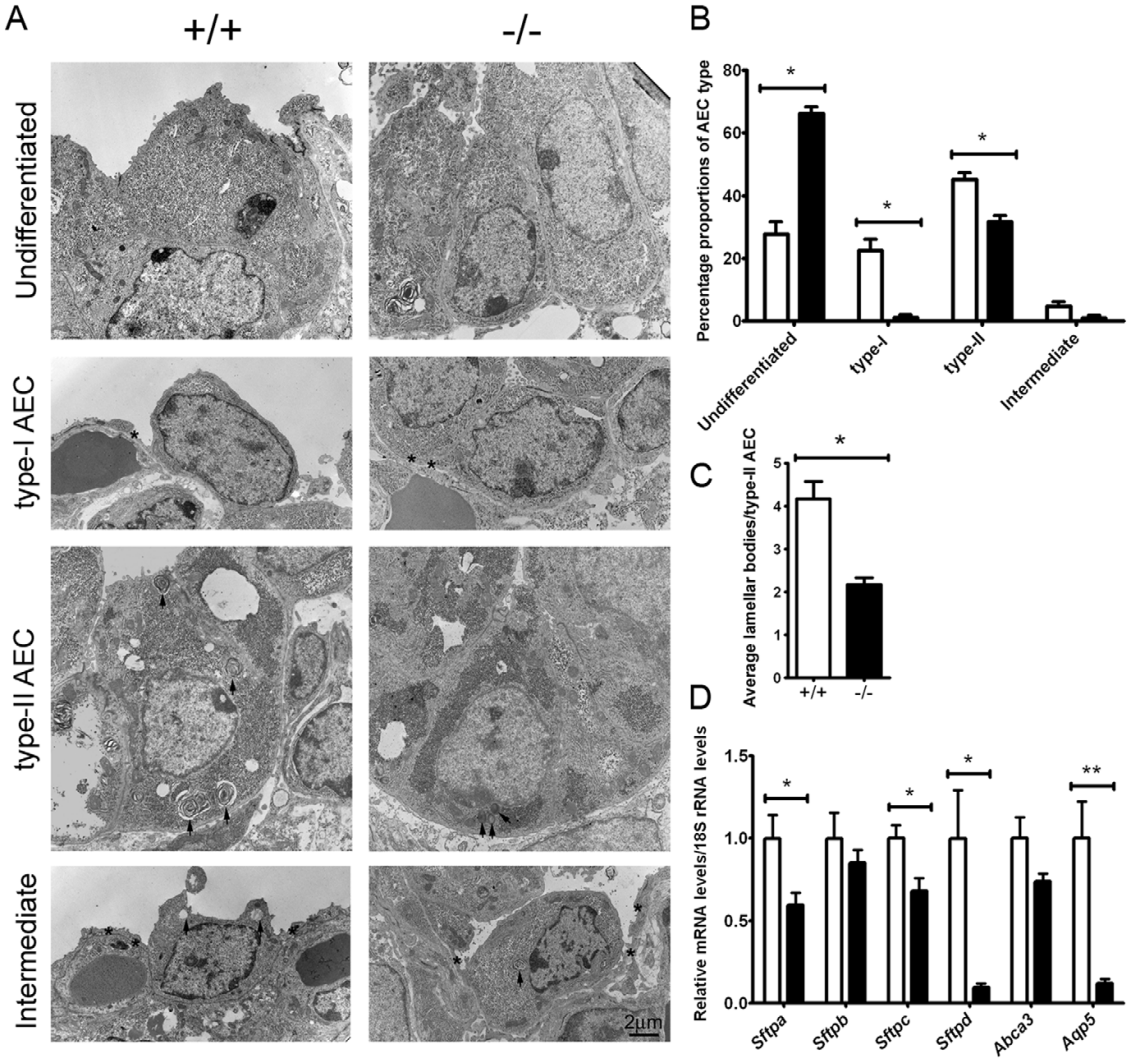
Defective AEC differentiation and lamellar body development in lungs of *Creb1* ^−/−^ mice. Tissue sections of E17.5 *Creb1* ^−/−^ and wildtype lungs were analysed for AEC proportions by electron microscopy. Representative electron micrographs show the ultrastructural appearance of undifferentiated, type-I, type-II and intermediate AECs in wildtype and *Creb1* ^−/−^ mice (A). Asterisks indicate cytoplasmic projections of the developing type-I AECs whereas arrows indicate lamellar bodies in type-II AECs. Note that the lamellar bodies in the type-II AEC from wildtype mice are more numerous and more mature in appearance when compared to lamellar bodies in *Creb1* ^−/−^ mice. Quantification of relative AEC proportions showed a statistically significant reduction in proportions of type-II and type-I AECs, together with a statistically significant increase in undifferentiated AECs (B). Quantification of lamellar body number showed a statistically significant reduction per cross sectional area of type-II AEC in E17.5 *Creb1* ^−/−^ (*n* = 4) lungs relative to wildtype (*n* = 5) (C). qPCR analysis of mRNA levels for *Sftpa*, *Sftpb*, *Sftpc*, *Sftpd* and also *Abca3* and *Aqp5* in the lung of E17.5 *Creb1* ^−/−^ mice relative to wildtype (*n* = 4). Error bars represent SEM. Asterisk (*) indicates *p*<0.05 and ** indicates *p*<0.001. *White bars*: Wildtype, *Black bars: Creb1* ^−/−^. Scale bars: 2 µm.

As activated Creb was found primarily in the distal lung epithelium, and that the onset of the *Creb1* ^−/−^ defect occurred at the developmental stage where AECs first begin to differentiate (E16.5–E17.5), we reasoned that an absence of Creb1 would have an adverse effect on AEC differentiation. In order to investigate this, the lungs of E17.5 *Creb1* ^−/−^ (*n* = 4) and wildtype (*n* = 5) mice were analysed using electron microscopy (EM) and the proportions of undifferentiated, type-I, type-II and intermediate AECs (Fig. 5A) were determined using strict morphological criteria (see methods). Although the proportion of type-II AECs was significantly reduced 1.4 fold in the *Creb1* ^−/−^ lung, proportions of type-I AECs were most dramatically affected with a 19 fold reduction in the *Creb1* ^−/−^ lung relative to wildtype (Fig. 5B). In contrast, the proportion of undifferentiated AECs was significantly increased by 2.4 fold in the *Creb1* ^−/−^ lung (Fig. 5B). The number of lamellar bodies per cross-sectional area of type-II AECs was also significantly reduced by 1.9 fold in the *Creb1* ^−/−^ lung (Fig. 4J), suggesting that type-II AECs in the *Creb1^−/−^* lung are relatively immature.

To further investigate AEC immaturity in *Creb1^−/−^* lungs we used qPCR to quantify mRNA levels of the surfactant protein genes: *Sftpa*, *Sftpb*, *Sftpc* and *Sftpd*, which are expressed within type-II AECs (Fig. 5D). We also quantified mRNA levels for *Abca3* (ATP-binding cassette sub-family A (ABC1), member 3) which encodes a factor required for lamellar body synthesis [25] as well as *Aqp5* (Aquaporin 5), a marker for type-I AECs (Fig. 5D). Levels of surfactant protein gene mRNA showed little or no differences in the *Creb1* ^−/−^ lung relative to wildtype, although the small reduction in *Sftpa* (1.7 fold) and *Sftpc* (1.5 fold) did reach statistical significance. The only exception to this was *Sftpd* which showed a large (10.6 fold) reduction in mRNA levels. Levels of *Abca3* mRNA were not significantly altered. qPCR analysis also found that *Aqp5* mRNA levels were significantly reduced (8.5 fold) relative to wildtype, which is consistent with the large reduction in type-I AEC proportions we observed in the *Creb1* ^−/−^ lung by EM analysis. These data suggest that differentiation of type-II AECs is mildly impaired in the *Creb1* ^−/−^ lung, however differentiation of type-I AECs is completely blocked.

### Sox9 and ProSPC are highly expressed in the distal epithelium of *Creb1* ^−/−^ lungs at late gestation

**Figure 6:**
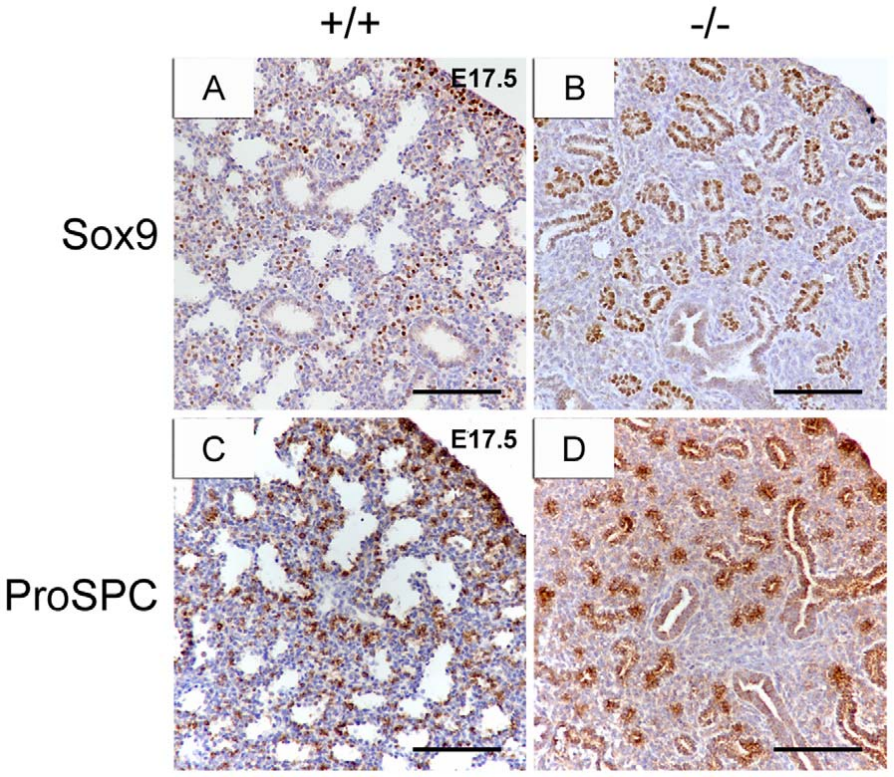
Sox9 and ProSPC are highly expressed in the distal lung epithelium of *Creb1* ^−/−^ mice. Immunohistochemistry for the epithelial progenitor cell markers Sox9 (A,B) and ProSPC (C,D) in the lung of E17.5 *Creb1* ^−/−^ and wildtype mice. Sox9 was detected sporadically in distal epithelial cells of wildtype lungs (A), but was found within almost all cells of distal epithelial tubules in *Creb1* ^−/−^ lungs (B). In wildtype lungs, ProSPC was detected only in type-II AECs (C), however almost all cells of the distal epithelium showed strong ProSPC expression in *Creb1* ^−/−^ lungs (B). Scale bars: 100 µm.

We further verified that loss of *Creb1* in the developing lung prevents normal differentiation of the distal epithelium. Sections of wildtype and *Creb1* ^−/−^ lungs were immunostained for the SRY-box containing gene 9 (Sox9) transcription factor, a marker of epithelial progenitor cells in the developing lung. Sox9 is expressed specifically in distal epithelial progenitor cells from E11.5 to E16.5, but is almost undetectable by E18.5 [1], [26]. We detected low levels of Sox9 in wildtype lungs at E17.5 (Fig. 6A). However in E17.5 *Creb1* ^−/−^ lungs, the distal epithelial tubules were almost entirely populated by Sox9-positive cells (Fig. 6B). Additionally, we stained wildtype and *Creb1* ^−/−^ lung sections for ProSPC, which is expressed in the lung epithelium at approximately E11.5 until E17.5 whereupon it becomes restricted to type-II AECs [27]. As expected, we detected ProSPC only in type-II AECs in E17.5 wildtype lung (Fig. 6C), however in E17.5 *Creb1* ^−/−^ lungs, ProSPC was found in almost all cells within distal epithelial tubules (Fig. 6D). Together, this suggests that the distal lung epithelium of *Creb1* ^−/−^ mice is almost entirely populated by undifferentiated progenitor cells at late gestation.

### Sftpd is localised to the developing conducting airway epithelium, but is absent in the *Creb1* ^−/−^ lung

**Figure 7:**
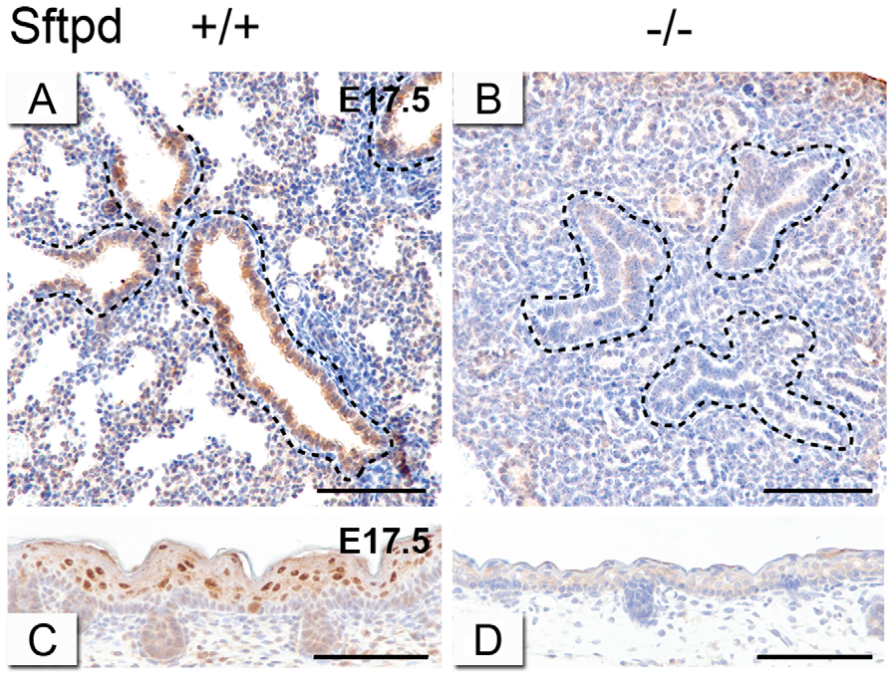
Sftpd is localised to the proximal airway epithelium, but is absent in the *Creb1* ^−/−^ lung. Immunohistochemistry for Sftpd protein in the lung (A,B) and epidermis (C,D) of E17.5 *Creb1* ^−/−^ and wildtype mice. Sftpd was localised primarily to the conducting airway epithelium (enclosed area) in the lung of wildtype mice (A), but was absent in the lung of *Creb1* ^−/−^ mice (B). Strong Sftpd expression was also detected in the stratum spinosum epidermal layer of wildtype (C), but not *Creb1* ^−/−^ (D) mice. Scale bars: 100 µm.

As *Sftpd* mRNA levels were particularly reduced in the E17.5 *Creb1* ^−/−^ lung compared with other surfactant protein genes, we investigated the localisation of Sftpd protein in wildtype lungs at E17.5 using immunohistochemistry and compared this to E17.5 *Creb1* ^−/−^ lungs. We detected only modest expression of Sftpd in the cytoplasm of wildtype conducting airway epithelium (Fig. 7A), however no expression could be seen in any compartment of the *Creb1* ^−/−^ lung (Fig. 7B). Interestingly, epidermal skin cells of the stratum spinosum layer showed strong Sftpd expression in E17.5 wildtype (Fig. 7C), but not E17.5 *Creb1* ^−/−^ (Fig. 7D) mice, suggesting that lack of Sftpd expression in *Creb1* ^−/−^ mice is not merely a secondary outcome of relative lung immaturity.

### Delayed expression of proximal epithelial cell markers in lungs of *Creb1* ^−/−^ fetal mice

**Figure 8:**
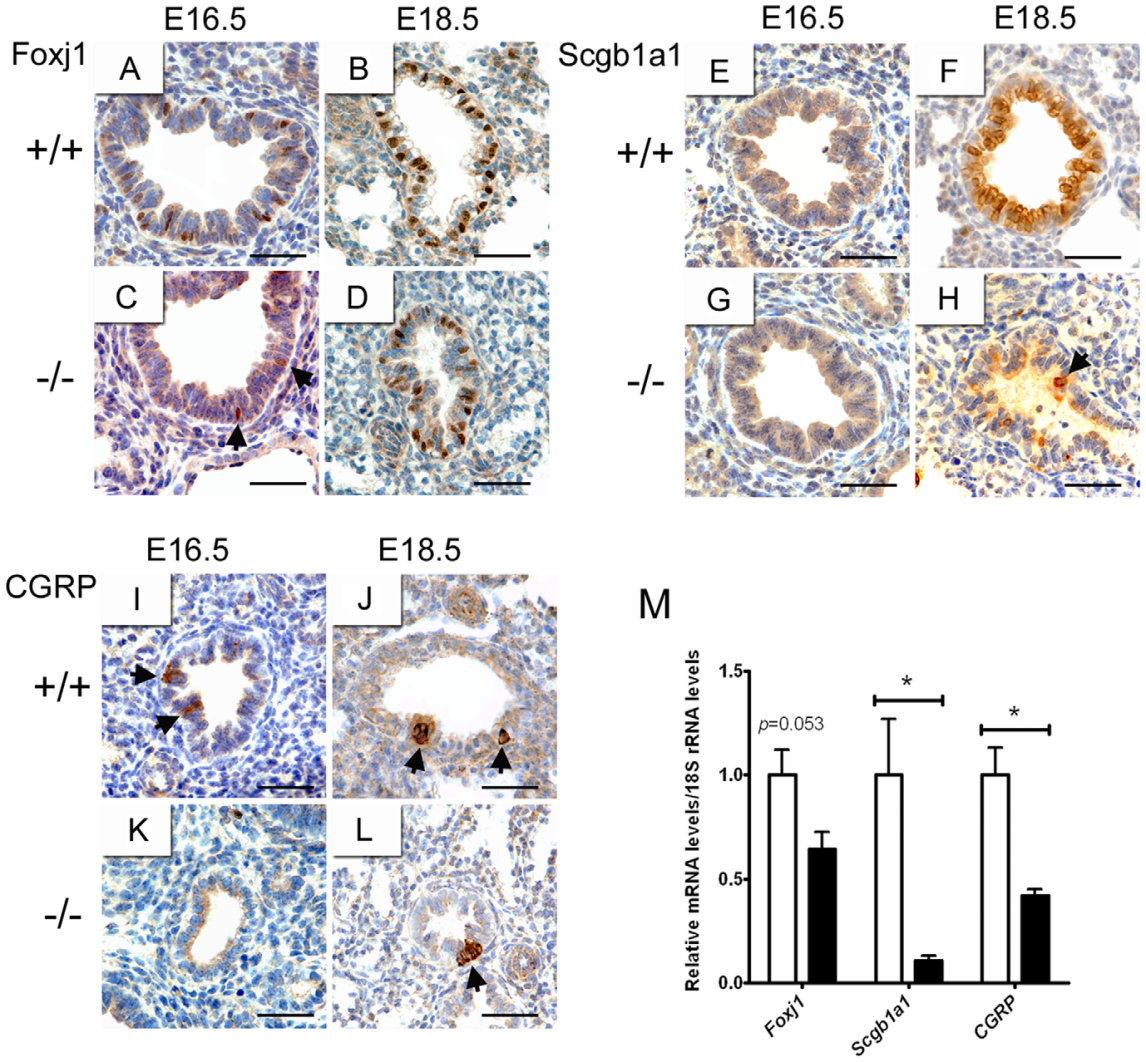
Delayed proximal epithelial cell differentiation in lungs of *Creb1* ^−/−^ fetal mice. Tissue sections from the lung of E16.5 and E18.5 *Creb1* ^−/−^ and wildtype were immunostained for Foxj1 (A–D), CC10 (E–H), and CGRP (I–L). At E16.5 Foxj1-positive cells were readily detected in conducting airway epithelium (A), but much less frequently in *Creb1* ^−/−^ lungs (C, arrowheads show two Foxj1-positive cells). Increased frequency of Foxj1-positive cells was detected in the lung of both E18.5 wildtype (B) and E18.5 *Creb1* ^−/−^ (D) mice. In wildtype lungs, Scgb1a1-positive cells were not detected at E16.5 (E,) but were common at E18.5 (F). In the lung of E18.5 *Creb1* ^−/−^ mice, Scgb1a1-positive cells were almost completely absent (H, arrowhead shows a solitary Scgb1a1-positive cell). Single CGRP-positive cells were detected at E16.5 in wildtype (I, arrowheads show CGRP-positive cells), but not *Creb1* ^−/−^ (K) lungs. At E18.5 Clusters of CGRP-positive cells were then detected in both wildtype and *Creb1* ^−/−^ lungs (J,L, arrowheads show CGRP-positive cell clusters). qPCR analysis of mRNA levels for *Foxj1*, *Scgb1a1* and *Calca* in the lung of E17.5 *Creb1* ^−/−^ mice relative to wildtype (*n* = 4)(M). Error bars represent SEM. Asterisk (*) indicates *p*<0.05. *White bars*: Wildtype, *Black bars: Creb1* ^−/−^. Scale bars: *A–L*, 50 µm.

Cell differentiation within the proximal airway epithelium was also examined in the lung of *Creb1* ^−/−^ fetal mice, using several specific immunomarkers of proximal airway epithelial cells. Scgb1a1 (also known as CC10, CCSP) is a marker of non-ciliated secretory Clara cells, Foxj1 (also known as HFH-4) is a marker of ciliated epithelial cells, and calcitonin gene related peptide (CGRP, also known as CALCA) is a marker of neuroepithelial cells. Using immunohistochemistry, lungs of E16.5 and E18.5 wildtype and *Creb1* ^−/−^ mice were analysed for expression of these markers as an indicator of proximal epithelial differentiation. Ciliated cells in the developing mouse lung first express Foxj1 at approximately E16.5 [28], and in lungs from E16.5 wildtype mice we detected a proportion of Foxj1-positive cells in the conducting epithelium (Fig. 8A) which increased in frequency at E18.5 (Fig. 8B). In the lung of *Creb1* ^−/−^ mice however, very few Foxj1-positive cells were detected at E16.5 (Fig. 8C), but were found at an increased frequency at E18.5 (Fig. 8D).

Clara cells first express Scgb1a1 at approximately E17.5 in developing mouse lung [27]. Consistent with this, we did not detect Scgb1a1-positive cells in E16.5 wildtype lung (Fig. 8E), but by E18.5 the majority of conducting airway epithelial cells expressed this marker (Fig. 8F). Lungs of *Creb1* ^−/−^ mice also showed no Scgb1a1-positive cells at E16.5 (Fig. 8G), however at E18.5 Scgb1a1-positive cells were almost completely absent (Fig. 8H). CGRP is first detected at E16.5 in the mouse lung [29]. Similarly, in wildtype lungs we detected single CGRP-positive cells in conducting airway epithelium at E16.5 (Fig. 8I), and then CGRP-positive clusters in E18.5 (Fig. 8J), indicative of neuroendocrine bodies (clusters of neuroendocrine cells). In contrast, we could not detect CGRP-positive cells in lungs of E16.5 *Creb1* ^−/−^ mice (Fig. 8K), but by E18.5 neuroendocrine bodies were detected (Fig. 8L). Levels of *Foxj1*, *Scgb1a1*, and *Calca* mRNAs in the lung of E17.5 *Creb1* ^−/−^ mice were also analysed using qPCR (*n* = 4) (Fig. 8M). *Scgb1a1* mRNA levels were markedly reduced (9.1 fold) in the *Creb1* ^−/−^ lung (*p*<0.05) and *Calca* mRNA levels were also reduced (2.4 fold, *p*<0.05). *FoxJ1* mRNA levels were reduced in the *Creb1* ^−/−^ lung (1.6 fold) though did not quite reach statistical significance (*p* = 0.053). Together, this indicates pulmonary ciliated and neuroendocrine cell differentiation may be delayed in the *Creb1* ^−/−^ lung while Clara cell differentiation is severely disrupted.

### Microarray analysis identifies potential Creb1-regulated genes in the developing mouse lung

To identify Creb1-regulated genes that may contribute to the observed defect at E17.5, total RNA from lungs of E17.5 wildtype and *Creb1* ^−/−^ mice (*n* = 4 for each) was analysed using Affymetrix mouse whole-genome expression exon microarray slides. A list of 694 genes was identified as being differentially expressed in lungs of *Creb1* ^−/−^ mice relative to wildtype; 416 gene targets showing reduced mRNA levels, and 278 gene targets showing increased mRNA levels. Investigation of the top ten most differentially expressed targets in the microarray list (Table 1) identified a number of genes known or suspected to be over- or under-expressed in the lungs of *Creb1* ^−/−^ fetal mice. These included: *Sftpd*, which we found under-expressed in the lung of E17.5 *Creb1* ^−/−^ mice as previously described (Fig. 5D and Fig. 7), *Scgb1a1*, which we found did not show evidence of protein expression until E18.5 in the *Creb1* ^−/−^ lung (Fig. 8), and *Crem*, which we found over-expressed in the lung of E17.5 *Creb1* ^−/−^ mice (Fig. 2A). Interestingly, a large proportion of the top ten most-highly under-expressed genes in the *Creb1* ^−/−^ lung are associated with a role in host defence and inflammatory response (*Chi3l1*, *Lyz1*, *Lcn2*, *Scgb1a1*, *Hc*, *Sftpd*, *Vnn1* and *H2-10*)(Table 1). Other highly under-expressed genes with known or predicted roles in immune response also appear with high frequency further down in our list including *Ier3* (12^th^), *Tnfaip* (14^th^), *Hp* (16^th^), *Lyz2* (21^st^) and *C3* (23^rd^). Over-expressed targets in the *Creb1* ^−/−^ lung however did not seem to belong to a particular ontology or function.

**Table 1:** Differentially expressed genes in the lung of E17.5 *Creb1* ^−/−^ mice identified by whole-genome microarray analysis.

Accession No.	Gene name	Fold change	*p* value	Function
NM_007695	Chitinase 3-like 1 (*Chi3l1*)	−41.3	0.0002	Inflammatory response
NM_013590	Lysozyme 1 (*Lyz1*)	−16.3	>0.0001	Defense response
NM_008491	Lipocalin 2 (*Lcn2*)	−11.3	0.0031	Inflammatory response
NM_011681	Secretoglobin, family 1A, member 1 (uteroglobin) (*Scgb1a1*)	−10.9	0.0013	Inflammatory response
NM_010406	Hemolytic complement (*Hc*)	−9.4	0.0002	Inflammatory response
NM_009160	Surfactant associated protein D (*Sftpd*)	−8.9	0.0001	Surfactant homeostasis
NM_009127	Stearoyl-Coenzyme A desaturase 1 (*Scd1*)	−8.5	0.0002	Fatty acid biosynthesis
NM_020509	Resistin like alpha (*Retnla*)	−7.1	0.0001	Regulation of apoptosis
NM_011704	Vanin 1 (*Vnn1*)	−6.3	0.0013	Inflammatory response
NM_010395	Histocompatibility 2, T region locus 10 (*H2-T10*)	−5.6	0.0036	Inflammatory response
NM_207161	cDNA sequence BC048355 (*BC048355*)	2.5	0.0008	Unknown
BC117501	RIKEN cDNA F630043A04 gene (*F630043A04Rik*)	2.5	0.0021	Unknown
NM_001110859	cAMP responsive element modulator (*Crem*)	2.7	>0.0001	Spermatogenesis
NM_027664	RIKEN cDNA 4933426K21 gene (*4933426K21Rik*)	2.8	>0.0001	Unknown
NM_021475	ADAM-like, decysin 1 (*Adamdec1*)	3.0	>0.0001	Integrin signalling pathway
NM_175499	SLIT and NTRK-like family, member 6 (*Slitrk6*)	3.1	0.0007	Axongenesis
NM_010733	leucine rich repeat protein 3, neuronal (*Lrrn3*)	3.3	0.0003	Endocytosis
AF084364	DNA segment, Chr 6, Miriam Meisler 5, expressed (*D6Mm5e*)	3.4	0.0001	Gametogenesis
NM_145073	histone cluster 1, H3g (*Hist1h3g*)	3.9	0.0037	Nucleosome assembly
NM_178216	histone cluster 2, H3c1 (*Hist2h3c1*)	4.8	0.0017	Nucleosome assembly

The top ten most-highly differential gene targets in the lung of E17.5 *Creb1* ^−/−^ mice are shown. Genes with lower mRNA expression in *Creb1* ^−/−^ lungs relative to wildtype (putative *Creb1*-induced) are listed first in descending order of fold change, while genes with higher mRNA expression in *Creb1* ^−/−^ lungs relative to wildtype (putative *Creb1*-repressed) are listed beneath in ascending order of fold change.

**Figure 9:**
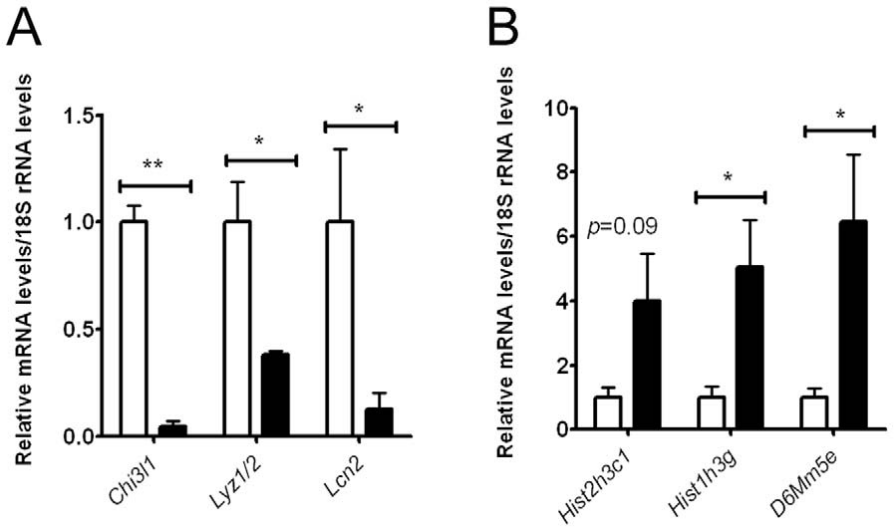
Gene expression analysis of highly-differential *Creb1* microarray lung gene targets. qPCR analysis of mRNA levels for down-regulated microarray gene targets: *Chi3l1*, *Lyz1/2*, and *Lcn2* in the lung of E17.5 *Creb1* ^−/−^ mice relative to wildtype (*n* = 4) (A). qPCR analysis of mRNA levels for up-regulated microarray gene targets: *Hist2h3c1*, *Hist1h3g*, and *D6Mm5e* in the lung of E17.5 *Creb1* ^−/−^ mice relative to wildtype (*n* = 4) (B). Error bars represent SEM. Asterisk (*) indicates *p*<0.05 and ** indicates *p*<0.001. *White bars*: Wildtype, *Black bars: Creb1* ^−/−^.

In order to validate our microarray data, we further investigated differential gene expression for the three most-highly down-regulated (*Chi3l1*, *Lyz1* and *Lcn2*) and up-regulated (*Hist2h3c1*, *Hist1h3g* and *D6Mm5e*) gene targets on our list in the lung of E17.5 *Creb1* ^−/−^ mice using qPCR (*n* = 4). Levels of *Chi3l1* and *Lcn2* mRNAs were reduced 12.5 fold (*p*<0.001) and 2.9 fold (*p*<0.05) in respectively (Fig. 9A). Due to very high sequence identity between *Lyz1* and *Lyz2* in mice, qPCR primers recognised *Lyz2* as well as *Lyz1*, and we detected a 2.6 fold (*p*<0.05) reduction in *Lyz1/2* levels in the *Creb1* ^−/−^ lung (Fig. 8A). Levels of *Hist2h3c1* mRNA were increased 4.0 fold in the *Creb1* ^−/−^ lung, but did not quite reach statistical significance (*p* = 0.09), while *Hist1h3g* and *D6Mm5e* mRNA levels increased 5.0 (*p*<0.05) and 6.5 fold (*p*<0.05) respectively, in the *Creb1* ^−/−^ lung (Fig. 9B). Taken together, these data support the validity of our microarray analysis.

## Discussion

A detailed analysis of the respiratory phenotype in fetal *Creb1* ^−/−^ mice has been performed and shows severely disrupted conducting and distal airway development, evident by E17.5. Furthermore, the timing of cell differentiation for a range of lung epithelial populations was strongly disrupted in lungs of *Creb1* ^−/−^ mice.

We first show via Ki67 and TUNEL immunostains that the respiratory defect in the lung of *Creb1* ^−/−^ mice is likely not correlated with a loss of cell proliferation or increased apoptosis, as might be expected from a lack of Creb1 during embryonic development. For example, loss of Creb1 specifically in chondrocytes reduces chondrocyte cell proliferation in mice at E14.5 to E17.5 [24] while loss of Creb1 specifically in the brain on a *Crem* ^−/−^ genetic background causes high levels of neuronal cell apoptosis at E18.5 [12]. Secondly, although we find that the lung from *Creb1* ^−/−^ mice are pale in colour, the respiratory defect we observe is unlikely to be due to impaired pulmonary circulation as we show that loss of Creb1 did not seem to affect development of the lung vasculature. This is further supported by a previous report which found no abnormalities in mice lacking Creb1 specifically in cardiomyocytes [30].

It is also important to note that the respiratory defect in *Creb1* ^−/−^ mice is unlikely to be merely a secondary outcome of general developmental retardation or arrest. For example, levels of surfactant protein mRNAs, with the exception of *Sftpd*, were not reduced in the lung of E17.5 *Creb1* ^−/−^ mice, whereas it would be expected that surfactant protein gene expression between a normal and a developmentally arrested fetus would be highly divergent, particularly considering the rapid increase in surfactant protein gene expression following type-II AEC differentiation at E17.5 [27], [31]. Additionally, a previous investigation indicated that the brain of E17.5 wildtype mice was clearly more developed than those of E18.5 *Creb1* ^−/−^ mice, despite their comparable size [11]. Therefore we conclude that the respiratory phenotype in *Creb1* ^−/−^ mice is caused directly by a loss of Creb1-mediated activity intrinsically within the developing lung.

This phenotype was demonstrated most clearly in the distal epithelium at E17.5, with severely disrupted differentiation of AECs. Differentiation of type-II AECs during lung development can occur either from early distal epithelial progenitors or via trans-differentiation from type-I AECs [14], [15]. In contrast, type-I AECs usually differentiate from a type-II AEC progenitor [32] but can also differentiate directly from distal epithelial progenitors as type-I AECs are often detected at an earlier developmental stage than type-II AECs [14], [33]. As the proportion of undifferentiated AECs remained high at E17.5 in the *Creb1* ^−/−^ lung, it is clear that Creb1 is required for both type-I and -II AEC differentiation from undifferentiated distal lung epithelial progenitor cells, though not absolutely required for type-II AEC differentiation. On the other hand, Creb1 function is essential for type-I AEC differentiation from both type-II AECs and undifferentiated distal epithelial progenitors.

We have also identified a role for Creb1 in the timing of differentiation of the proximal lung epithelium. Immunomarkers for the ciliated (Foxj1), non-ciliated secretory Clara (Scgb1a1) and neuroendocrine (CGRP) cell populations, which together constitute the majority of the conducting airway epithelium, all show delayed appearance in the lung of *Creb1* ^−/−^ mice. Creb1 may therefore mediate differentiation throughout the entire conducting airway epithelium though this needs to shown for other epithelial populations such as goblet and basal cells, which appear in the upper bronchial and tracheal epithelium. It is also evident that increased differentiation of Foxj1-, Scgb1a1-, and CGRP-positive cells continues to occur beyond E17.5 in lungs of *Creb1* ^−/−^ mice when structural maturation of lungs seems to be arrested. This invites the possibility that other epithelial populations, such as AECs could further differentiate after E18.5 in the *Creb1* ^−/−^ lung, however as *Creb1* ^−/−^ mice die at birth this is currently impossible to investigate.

The mechanism by which Creb1 contributes to structural maturation or epithelial differentiation in the developing lung is unclear. As phosphorylation of Creb1 was detected primarily in distal epithelium at E18.5, and not in surrounding mesenchyme until E18.5, it is likely that Creb1 mediates gene expression in a cell-autonomous manner to induce differentiation or specify the cell fate of distal epithelial populations, such as AECs, rather than via signalling from the adjacent mesenchyme. Indeed, the lack of Creb1 expression we observed in the mesenchyme surrounding the epithelium at E16.5 may be a means to prevent activation of Creb1. However it is also likely that Creb1-mediated signalling in the distal epithelium is not required or much utilized after E16.5 as at E18.5, pCreb1 was localised primarily to the mesenchymal cells within saccular walls. Creb1 signalling from the mesenchyme beyond E18.5 may therefore have an important role in postnatal lung development. Although we find differentiation of proximal epithelium is also affected in lungs of *Creb1* ^−/−^ mice, we detected only sparse activation of Creb1 in these cells. We suggest that perhaps Creb1 is activated in these cells much earlier for a specific interval to promote cell differentiation, and then activation is repressed in the same way as pCreb1 is eventually lost in distal epithelium by E18.5. Transient activation of Creb1 by phosphorylation has also been reported in mouse neuroblasts during neuronal differentiation [34].

The genes regulated by Creb1 in the developing lung are almost completely unknown. Several studies have shown that cAMP stimulates gene expression of surfactant protein mRNAs [35] however Creb1 can be activated by signalling pathways other than those which are stimulated by cAMP. Our microarray analysis and qPCR data at E17.5 indicate that with the exception of *Sftpd*, surfactant protein mRNAs are not greatly affected by a loss of Creb1. Sftpd, together with Sftpa1 and serum mannose-binding protein are members of the calcium-dependent collagenous lectin (collectin) subfamily of mammalian C-type, lectins, and is involved with the immune response to inhaled pathogens and microorganisms [36]. *Sftpd* mRNA is initially expressed at E16 in mice [37], and its expression is limited to type-II AECs and non-ciliated bronchiolar epithelial cells which are most likely Clara cells [38], [39]. In mice, we show Sftpd protein is localised to the conducting airway epithelium at E17.5. It is therefore possible that the very large reduction in *Sftpd* mRNA levels and also lack of Sftpd protein immunostaining in the *Creb1* ^−/−^ lung we observe at E17.5 may be due to the absence of Scgb1a1-positive Clara cells which we detect at this stage of development. Alternatively Creb1 may have an active role in *Sftpd* gene transcription since we also show a loss of Sftpd expression in non-lung tissues such as the stratum spinosum epidermal layer in E17.5 *Creb1* ^−/−^ mice compared with robust expression in wildtype mice. Transcriptional regulation of the *Sftpd* promoter has not been thoroughly explored, however studies to date in human and mouse have revealed a role for AP-1 proteins [40], CCAAT enhancer-binding proteins (C/EBPs) [41] and Nuclear factor of activated T cells (NFATs) [42] in directing *Sftpd* gene expression.

As well as *Sftpd*, the microarray analysis detected a high number of genes involved with the immune response such as *Chi3l1*, *Lyz1*&*2*, *Lcn2*, *Scgb1a1*, *Hc*, *Vnn1*, *H2-10*, *Ier3*, *Tnfaip*, *Hp* and *C3* which are also highly under-expressed in the E17.5 *Creb1* ^−/−^ lung. This suggests a role for Creb1 in stimulating expression of genes required for the regulation of the immune response in the developing lung. For example, Chi3l1 promotes T cell helper 2 (Th2) -induced inflammation in response to an allergen challenge [43], Hc and C3 together constitute crucial elements of the complement system which mediates several immune responses to target foreign pathogens [44], while Lcn2 [45] and Lyz factors [46] have important roles in the immune response to bacterial infection. How Creb1-mediated stimulation of immune response-associated genes is related to development of the lung is unclear, however interestingly the induction of pro-inflammatory mediators has been correlated with increased maturation of the fetal lung close to term, and may even be a more potent accelerator of lung development than corticosteroid exposure [47]. Furthermore, very similar trends in expression of immune response-related genes has also been shown in the lungs of mice with targeted mutations of *Nkx2-1* [48], *Foxa2* [49], *C/ebpα* [50] and *Cnb1* [51], all of which exhibit a severe disruption in lung development at late gestation, similar to *Creb1* ^−/−^ mice. Microarray analyses of fetal lungs performed in these studies show that expression of genes involved in the immune response and host defence are among the most highly differential, usually with reduced expression compared to wildtype . It is even likely that *Lyz1* and *Lyz2* are direct transcriptional targets of Foxa2 [49]. Further studies are therefore needed to investigate the clear link between transcription factor signalling and genes involved with immune response in the context of lung development.

In conclusion, we show that Creb1-mediated signalling is required for proper differentiation of important epithelial cell populations in the developing lung including ciliated, Clara, and neuroendocrine cells in the proximal airway, as well as type-II and particularly type-I AECs of the distal epithelium. Further investigation will be needed to determine how the timing of Creb1 phosphorylation in lung cell populations affect epithelial differentiation, both before and after birth, and also which genes are regulated by Creb1 in the developing lung to promote its effects. It will be particularly interesting to determine the effects of a cell-type specific targeted deletion of Creb1 in the respiratory epithelium. Additionally, it will be useful to identify the upstream signalling pathways responsible for phosphorylation of Creb1 specifically in the developing lung, and also which circulating ligands stimulate these pathways.

## Supporting Information

Figure S1 **Creb1 protein is not detected in the lung of** ***Creb1*** **^−/−^ mice.** Immunohistochemistry for Creb1 protein in the lung of E18.5 wildtype and *Creb1* ^−/−^ mice. Creb1 is strongly detected in the lung of wildtype mice (A), but is almost completely absent in the lung of *Creb1* ^−/−^ mice (B). Scale bars: 100 µm.
(TIF)

Table S1 **Analysis of** ***Creb1*** **^−/−^ mouse mortality in utero.**
(DOC)
