# Correction: cAMP Response Element Binding Protein Is Required for Differentiation of Respiratory Epithelium during Murine Development

**DOI:** 10.1371/annotation/ec04ad74-63cc-4fbe-9ad8-074a1d62fdf4

**Published:** 2012-01-27

**Authors:** A. Daniel Bird, Sharon J. Flecknoe, Kheng H. Tan, P. Fredrik Olsson, Nisha Antony, Theo Mantamadiotis, Richard Mollard, Stuart B. Hooper, Timothy J. Cole

An author was omitted from the author list. Richard Mollard should be listed as seventh author. His affiliation is 1 Department of Biochemistry & Molecular Biology, Monash University, Clayton, Victoria, Australia. His author contributions are: Analyzed the data, wrote the manuscript.

