# Supplementary figures and images for: cAMP Response Element Binding Protein Is Required for Differentiation of Respiratory Epithelium during Murine Development
Source: PLoS One. 2011 Mar 8;6(3):e17843. doi: 10.1371/journal.pone.0017843 (PMC3050929; doi:10.1371/journal.pone.0017843)

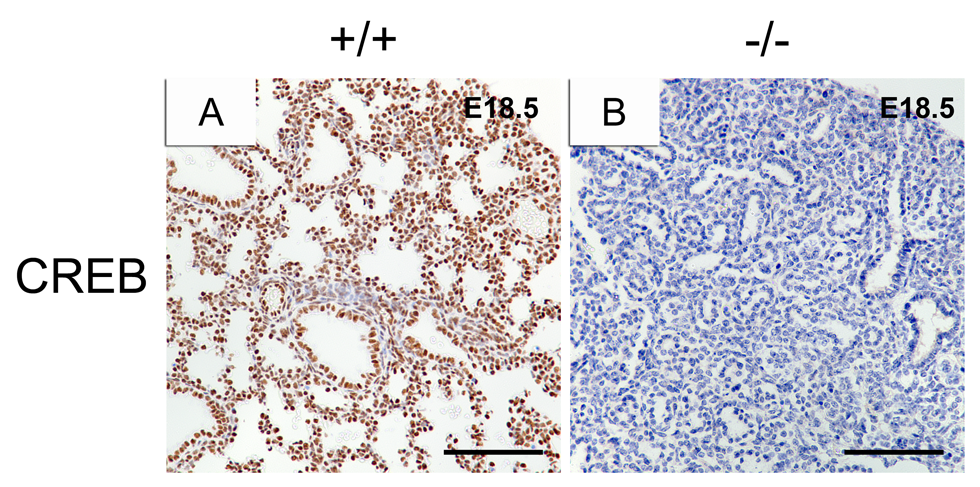

Supplement: Figure S1 — Creb1 protein is not detected in the lung of Creb1 −/− mice. Immunohistochemistry for Creb1 protein in the lung of E18.5 wildtype and Creb1 −/− mice. Creb1 is strongly detected in the lung of wildtype mice (A), but is almost completely absent in the lung of Creb1 −/− mice (B). Scale bars: 100 µm. (TIF) [file pone.0017843.s001.tif]
