# Supplementary material for: cAMP Response Element Binding Protein Is Required for Differentiation of Respiratory Epithelium during Murine Development
Source: PLoS One. 2011 Mar 8;6(3):e17843. doi: 10.1371/journal.pone.0017843 (PMC3050929; doi:10.1371/journal.pone.0017843)
Supplement: Table S1 — Analysis of Creb1 −/− mouse mortality in utero. (DOC) [file pone.0017843.s002.doc]

**Supporting Table 1: Analysis of *Creb1*^-/-^** mouse mortality in utero.

| **Gestational age** | **Average % *Creb1*^-/-^ mice/litter** | **#litters** | **# total pups** |
| --- | --- | --- | --- |
| E15.5 | 12.7 | 7 | 52 |
| E16.5 | 9.0 | 5 | 32 |
| E17.5 | 6.7 | 50 | 323 |
| E18.5 | 8.7 | 25 | 151 |

Proportions of *Creb1*^-/-^ mice per litter were calculated for E15.5 to E18.5. As expected, proportions of *Creb1*^-/-^ mice were present at a reduced Mendelian frequency.
